# The Implementation of Teleconsultations in a Physiotherapy Service During Covid-19 Pandemic in Brazil: a Case Report

**DOI:** 10.5195/ijt.2021.6368

**Published:** 2021-06-22

**Authors:** Thaiana B. F. Pacheco, Dayse A. Bezerra, João Pedro de S. Silva, Ênio W. A. Cacho, Clécio G. de Souza, Roberta O. Cacho

**Affiliations:** 1 Faculty of Health Sciences of Trairí, Federal University of Rio Grande Do Norte, Santa Cruz, Rio Grande Do Norte, Brazil

**Keywords:** COVID-19, Physiotherapy, Telehealth, Telerehabilitation

## Abstract

**Introduction:**

The Brazilian Council of Physiotherapy and Occupational Therapy regulated the use of teleconsultation during the COVID-19 pandemic, creating uncertainty about its use in Brazil.

**Objective:**

To describe the experience of teleconsultations during the COVID-19 pandemic.

**Methods:**

Four patients participated in the study with the following diagnoses: Parkinson's disease, stroke, peripheral facial paralysis, and tibial plateau fracture. Patients underwent up to 10 physiotherapy sessions via digital tools. The 5-A self-management tool (Assess, Advise, Agree, Assist, Arrange) guided the sessions.

**Results:**

The teleconsultation type varied between synchronous (n = 1; 25%); asynchronous (n = 2; 50%) and synchronous/asynchronous (n = 1; 25%). There was 75% (n = 3) adherence and one withdrawal (25%). As the benefits of teleconsultations, the patients pointed out the convenience, maintenance of the exercises, and contact with the professional. The reported limitations were the lack of the use of physiotherapeutic devices.

**Conclusion:**

Teleconsultations contribute to the continuity of physiotherapy treatment during social isolation. Adherence to treatment was facilitated by access to the technology and by offering patients the choice of teleconsultation type.

Beginning on March 23, 2020, the Brazilian Council of Physiotherapy and Occupational Therapy (COFFITO), regulated the modalities of teleconsultation, telemonitoring, and teleconsulting in response to the crisis caused by the new coronavirus pandemic (COFFITO, 2020). With the adoption of social distancing, clinics were closed and thus, this regulation aimed to establish strategies that would prioritize the care of patients who needed to continue their treatment without having their functional prognosis negatively impacted by social distancing. Continuity of care was especially important for individuals with chronic, inflammatory or progressive diseases, and those whose care depended upon a critical therapeutic window for rehabilitation (i.e., postoperative and acute phases of cerebrovascular events or traumas). The COFFITO also aimed to mitigate the economic impact of the pandemic on professionals.

Social distancing can increase the restrictions on mobility during performance of daily living activities, leading to greater functional dependence. Individuals in need of continued rehabilitation are most affected by these limitations. Thus, in addition to providing remote physical therapy, telerehabilitation stratgies should be planned that encourage the motivation of unsupervised therapeutic practice, self-management, and patient-centered treatments.

Telereahabilitation is a method of service delivery that employs telecommunication technologies to connect patients and rehabilitation professionals at a distance. Depending upon the tool used in telerehabilitation, the consultation can be characterized as synchronous (i.e., the professional and patient are available at the same time, as in audio and video calls), or asynchronous (i.e., interactions between professional and patient do not occur in real time). Examples of asynchronous telerehabilitation methods are e-mails, text messages, and recordings of images, videos and audios that the patient or practitioner are able to consult at a different time (Souza-Junior, 2014).

Facing the novel coronavirus pandemic, groups of researchers have been developing scientific content to guide professionals in the practice of telerehabilitation. The Chartered Society of Physiotherapy, for example, developed quick guides with practical guidance on how to implement telerehabilitation and what digital tools should be used to address different clinical needs (The Chartered Society of Physiotherapy, 2020).

However, even before the pandemic, telerehabilitation was investigated in the scientific community and reports on its effectiveness were already known. Systematic reviews have shown similar effects between the use of telerehabilitation and conventional therapies in studies involving patients with different motor dysfunctions (Agostini et al., 2015), stroke (Laver et al., 2020) and in orthopedic postoperative conditions (Pastora-Bernal et al., 2017).

Although there are numerous studies on telerehabilitation, this modality was first allowed for physiotherapy in Brazil as a response to the pandemic. There is currently uncertainty about the effectivenss of telerehabilitation in the Brazilian population, and whether it will continue to be allowed post-pandemic. Thus, the aim of this study is to report the experience of telerehabilitation during the social distancing caused by the novel COVID-19 pandemic.

## METHODS

This is a report of cases that describes the progress of four patients who were treated by the School of Physical Therapy of the Faculty of Health Sciences of Trairi (FACISA) before the social distancing recommendation and then started to receive care via telerehabiliation during the pandemic. FACISA is a specialized academic unit at the Federal University of Rio Grande do Norte (UFRN) located in the city of Santa Cruz/RN, Brazil. In June of 2020, the epidemiological bulletin reported 15,985 confirmed cases of COVID-19 in the State of Rio Grande do Norte, with an incidence of COVID-19 in 254.6 per 100,000 inhabitants of Santa Cruz. This reinforces the importance of telerehabilitation to maintain social distancing in this region.

This study was carried out within the rules of the Research Ethics Committee of UFRN under opinion number 4.100.991 and in accordance with the resolution 510/16 of the National Health Council. All participants involved signed the Informed Consent Term, authorizing their voluntary participation. Four patients with the following diagnoses participated in this study: Parkinson's disease (case 01), stroke (case 02), peripheral facial palsy (case 03) and tibial plateau fracture (case 04). To be eligible for telerehabilitation, patients met any of the following criteria: (1) diagnosed with a progressive disease or in an acute or subacute post-ictus phase; (2) access to some digital communication tool with internet access (e.g., smartphone, notebook, tablet).

First, clinical and sociodemographic data were extracted from patients' medical records. Then, two physiotherapists from FACISA conducted teleconsultation to patients' homes, using their own resources to make the calls.

The communication tool used in each case was chosen considering the ease of access and management by patients, varying between explanatory audios or videos, images, and video calls. The teleconsultations began in the second week of March 2020, making a total of up to 10 sessions distributed at a frequency that varied according to each patient.

The data were reported descriptively considering the adherence to telerehabilitation, the proposed therapeutic plan, and the components of the 5-A model for health self-management (Assess, Advise, Agree, Assist, Arrange) that aims to develop an individualized action plan based on behavioral changes in the health context (Glasgow et al., 2003).

## CASE DESCRIPTIONS

## CASE 01

A 69 year-old man, with the diagnosis of Parkinson's disease since 2010, had completed high school and was retired from employment. He complained of a lack of resistance and fatigue in his lower limbs. On his last physical in-person examination, a balance deficit was observed, with a score of 41 points on the Berg Balance Scale (Scalzo et al., 2009). The patient presented with impaired gait, needing crutches or a walker for locomotion. He performed the Timed up and Go test (Rosa et al., 2017) in 40.93 seconds with a walking speed of 0.14m/s. The Freezing Gait Scale (Baggio et al., 2012) scored 20. He had been assisted at FACISA's School of Physical Therapy clinic since 2013, three times a week for 50 minutes each session.

The patient's telerehabilitation protocol occurred during 10 teleconsultations using audio sharing in Whatsapp. The patient opted to use audio sharing because he considered that easier and felt more independent. The therapeutic plan was sent in the form of recorded audios and photos with exercise models, and the monitoring was performed asynchronously. The first contact by phone took place on March 23, 2020. At the time, the patient was surprised by the new type of assistance and did not immediately understand the proposal for telerehabilitation. The structure of each teleconsultation session consisted of the following steps: (1) response of the patient to the execution of the exercises; (2) motivational speech to the patient to establish the exercise routine; (3) prescribing new exercises; (4) adaptations to the exercise (if necessary); and (5) monitoring the patient using the BORG scale (Cabral et al., 2017).

The frequency of the teleconsultations varied between two to three times a week. The patient provided daily feedbacks about the execution of the exercises, as well as about his performance.

## CASE 02

A 70 year-old man with an incomplete elementary school education was admitted at FACISA'a School of Physical Therapy in March 2020 with right hemiparesis and slow gait caused by ischemic stroke which had occurred in December 2019. As the patient did not have his own digital tool and did not know how to use one, the teleconsultations were mediated by the patient's son and carried out asynchronously through text messages and áudio sharing in Whatsapp.

The lack of direct contact with the patient led to a communication barrier, increasing the interval between the contacts. The first teleconsultation occurred on March 30th and the first feedback was received on April 6, in which the son reported that the father had the flu (Table 2). In addition, the son made the following report: “He speaks with difficulty but he is starting to improve his voice … and about walking … he walks a little with the walker… he sits in the veranda, watches people pass by on the street, he sits down to eat meals. He likes to stay lying down and I sometimes agree so he does not get nervous …”.

Facing this report, we reinforced the importance of keeping the patient active, and encouraged sedestation, interaction with the family, communication, and the importance of encouraging movements in this moment of social distance. After this orientation, communication was attempted until April 14, 2020 without success.

## CASE 03

A 55 year-old woman, with a complete elementary school education, resided in the city of Santa Cruz as a housewife. She was admitted at FACISA School of Physical Therapy on January 22, 2020, ten days after receiving a clinical diagnosis of Peripheral Facial Palsy. She reported difficulty in keeping food inside of her mouth. Physical examination revealed hypotonia in the left hemiface, facial asymmetry and the presence of Bell's sign, Tinel's sign, lagophthalmos and sialorrhea. In the muscular function test, there was a generalized weakness of the left hemiface graded between 0 and 2, presenting grade 3 only for the buccinator muscle. The telerehabilitation began on March 26, 2020 with the patient showing strength grade 4 for all the muscles of the left hemiface, except for the zygomatic, which had grade 2. Teleconsultations were carried out once a week in both modalities: synchronous and asynchronous. The synchronous teleconsultation occurred through 30-minute video calls, in which the exercises were prescribed by the therapist and performed by the patient, with remote supervision. For the asynchronous modality, audio and text messages were shared, in which the therapist motivated the patient to maintain the exercise routine. In addition, the patient provided photos showing the performance of the exercises and the evolution of her functional condition.

## CASE 04

A 32 year-old woman, a nurse with postgraduate education, was a crossfit practitioner. She was admitted to FACISA's School of Physical Therapy on November 26, 2019 with the diagnosis of left tibial plateau fracture due to a motorcycle accident that occurred on November 10, 2019. At the time of admission, she reported loss of strength and muscle atrophy in the left thigh and pain and swelling in the left knee. Before the pandemic, the patient was treated twice a week for 50 minute sessions in which she progressed with independent gait and muscle strengthening and proprioceptive training.

The telerehabilitation protocol for this patient started on March 24, 2020 and consisted of 10 video calls through Whatsapp, twice a week with mean duration of 50 minutes. The exercises proposed were planned in order to maintain the gains achieved throughout the prior in-person sessions, as well as to progress and improve the kinetic and functional conditions, to restore the complete range of motion of the left knee, and to recover the complete muscle strength in the left lower limb. The patient showed good adherence to telerehabilitation. Her motivation and level of functional independence, and the quality of the internet contributed to the success of the sessions.

## RESULTS

Table 1 shows the characterization of the participants included in the study. Three participants had a diagnosis of neurological disease and one of orthopedic trauma. Fifty percent were older men and 50% adult female. The teleconsultation modalities varied according to the profile of each patient: entirely asynchronous, fully synchronous, or a combination of both.

**Table 1:** Characterization of Participants

Case	Age	Sex	Diagnosis	Telerehabilitation modality
01	69	M	Parkinson's Disease	Asynchronous
02	70	M	Stroke	Asynchronous
03	55	F	Peripheral Facial Palsy	Synchronous/asynchronous
04	32	F	Tibial plateau fracture	Synchronous

*Note.* M: Male; F: Female.

Source: research data.

Table 2 shows the categorization of the telerehabilitation considering the 5-A model for health self-management.

**Table 2:** 5-A model application in telerehabilitation strategy

5-A model	Case 01	Case 02	Case 03	Case 04
**Assess**	Questioning about facilitators and barriers for telerehabilitation.	Assessment of the possibility of using the family member's cell phone.	Questioning about facilitators and barriers for telerehabilitation.	Questioning about facilitators and barriers for telerehabilitation.
**Advise**	Sharing educational material on Parkinson's Disease	Explanation on the importance of exercises for the patient's functional mobility.	Explanations about the diagnosis, disease progression, and the benefits of continuing treatment and maintaining social isolation.	Sharing information about the patient's functional condition.
**Agree**	Two options of exercise for the same therapeutic goal.	Motivational speeches for the family member to establish the exercise routine with the patient.	Goal setting based on the patient's clinical needs and choices.	Goal setting based on the patient's clinical needs and choices.
**Assist**	Identify equipment the patient could have at home to assist in telerehabilitation.	Use of cell phone to share information.	Use of cell phone to share information.	Identify equipment the patient could have at home to assist in telerehabilitation.
**Arrange**	Use of BORG scale for monitoring.	Photos of the exercises were requested, but no feedback was provided.	Sharing of photos.	Patient's feedback.

At the end of the intervention period, patients were able to report their individual perceptions about the telerehabilitation, both facilitating factors and barriers:

“Even at a distance, this monitoring is very important because the patient keeps doing his exercises regularly, alleviating the problems and suffering that we have” (Case 01).

“I miss the devices and the guidance that the school staff provides (professor, students, etc.). We do an exercise that doesn’t work and the professor corrects it. This gives a lot of confidence to the patient. And the devices… we don’t have the devices you have at the clinic, so I miss the devices. But the positive points surpass all of that and we will transforming this absence in willpower and overcome everything that we are going through…” (Case 01).

“It is great to have someone who will accompany you even if it is on video. I liked it a lot because it indicates that the “doctor” is interested in the patient. And I, as a patient, can only be grateful. I mean, it didn't hurt me at all, quite the contrary. I really liked that you were interested in calling me and helping me to do the exercises correctly. You see that my improvement was great and then you asked me to continue only two exercises because the rest of my face is getting better” (Case 03).

“Convenience to be attended at home, not having to interrupt the service and having to spend a long time without physical therapy” (Case 04).

“The difficulties that I see can be overcome. The issue of equipment that I do not have at home, but can be replaced with the weight of the body itself. Another difficulty that I may have, but that I haven’t had yet, is internet problems that can happen affecting the calls at the scheduled time” (Case 04).

## DISCUSSION

This study aimed to describe the experience of the telerehabilitation in the current period of social isolation. There was good adherence to the proposed treatment modality, since 75% of the participants continued the treatment.

The search for effective and motivating strategies that facilitate the continuity and adherence to treatment has always been a great challenge for physical therapists. However, the current situation of social isolation, experienced all over the world, raises the following challenge: how to provide effective and motivating physiotherapeutic care while respecting social distancing? It is with this perspective that digital tools can be combined with therapeutic practices and help patients remotely, keeping them motivated and active.

Although telerehabilitation is a service delivery model widely studied and applied in countries all over the world, the physical therapy practice in Brazil still reflects a culture of direct and in-person contact, in which the responsibility for the patient's kinetic and functional prognosis is most often centered on the physiotherapist. As a result, the patients' reports that the absence of therapeutic equipment was a limiting factor during telerehabilitation may reflect the preconception that assistance within the clinical environment is more determinant of the patient's kinetic and functional prognosis than the establishment of a routine of remote therapeutic exercises.

With this in mind, the 5-A model (Assess, Advise, Agree, Assist, Arrange) has been used as a strategy to develop therapeutic plans centered on the patient and based on health-related behavior. The 5-A model supports changes in attitudes that impact the patient's health and enhances self-management skills (Siegert et al., 2019).

Although the 5-A model has not been developed specifically for telerehabilitation, the implementation of its concepts in telehealth services can contribute to the development of a patient-centered practice, in which patients become part of their rehabilitation process and assume a commitment to performing the exercises at home. Considering the telerehabilitation context, the experience reported in the present study integrated some strategies (Morais et al., 2015) that are related to the 5-A model, such as: assessing the ability of patients to perform a computational intervention for self-management (Assess); intervention program with the objective of involving the patient in self-care (Advise); determining options (Agree); interventions with the use of technological resources (Assist); and monitoring the self-management plan (Arrange).

The benefits attributed to rehabilitation are diverse and widely known. Individuals with Parkinson's disease, for example, show a 25% improvement in activities related to mobility and self-care when they are involved in rehabilitation programs and are motivated to perform exercises on a regular basis, even if in an adapted way (Tickle-Degnen et al., 2011). The need for telerehabilitation based adaptations to exercises can challenge patients’ motivations. In contrast, for the physiotherapist, establishing a therapeutic plan in teleconsultation can be extremely enriching, since it is an opportunity to see the patient within their home context, making it possible to understand their family interpersonal relationships and observe emotional and environmental factors that can mitigate barriers and identify facilitating components.

It is important to note that among the cases reported in this study, we did not obtain 100% adherence to telerehabilitation. Although studies point to telerehabilitation as a tool that can remedy the lack of access of part of the population to health care networks, limited access to telecommunication tools can restrict the usability of telerehabilitation in groups of people that reside in regions where there is a considerably low level of education and income and a restricted telephone and internet network (Cottrell et al., 2016). Such was the case for the nurse living in Santa Cruz (Case 04). This observation is consistent with the study that cited the lack of flexibility of devices, and the unsatisfactory coverage in the connectivity networks as limitations for the wide application of telerehabilitation (Agostini et al., 2015).

Besides the connectivity difficulties mentioned above, guidelines from the Chartered Society of Physiotherapy on the implementation of teleconsultations during the COVID-19 pandemic suggested that some competencies and skills required training and/or previous experience for the therapists (e.g., attenuation of distractions; behavioral practices focused on the therapeutic context; flexible schedules and self-management). In addition, training on the use of communication technologies would enable patients and family members to better understand and participate in the virtual experience of telerehabilitation, as would providing parallel institutional communication platforms. Such strategies could contribute to patient compliance with the telerehabilitation program.

The present study highlights the application of telerehabilitation during a period of social isolation. Although COFFITO's regulation is limited to a strategy to deal with the new coronavirus pandemic, the present investigation of the use of teleconsultation suggests future uses of telerehabilitation as a tool that could be combined with conventional therapeutic practices.

This series of cases demonstrated the versatility of telerehabilitation in treating different clinical conditions, such heterogeneity limits the understanding of the role of telerehabilitation in terms of adherence to physical therapy treatment. In addition, the present study was developed with researchers' resources, which might not approximate the actual range of digital devices that could contribute to patient motivation and adherence to remote treatment.

## CONCLUSION

We concluded that telerehabilitation is a modality that can contribute to the maintaining of physical therapy treatment in the face of social distancing. The patients' access and knowledge concerning communication tools and the strategy chosen by the professionals favor better adherence to telerehabilitation.
